# High uPAR and Low miR-221 Expression Predict Poor Disease-Free Survival in Triple-Negative Breast Cancer

**DOI:** 10.3390/pathophysiology33020029

**Published:** 2026-04-22

**Authors:** Weiwei Gong, Yueyang Liu, Natalie Falkenberg, Marion Kiechle, Holger Bronger, Julia Dorn, Viktor Magdolen, Tobias Dreyer

**Affiliations:** 1 Department of Hematology and Oncology, Guangzhou Women and Children’s Medical Center, Guangzhou Medical University, Guangzhou 510080, China; gongweiwei@gwcmc.org; 2 Department of Gynecology, Guangdong Provincial People’s Hospital (Guangdong Academy of Medical Sciences), Southern Medical University, Guangzhou 510080, China; liuyueyang@gdph.org.cn; 3 Department of Obstetrics and Gynecology, Technical University of Munich, 81675 Munich, Germany; natalie.falkenberg@gmx.de (N.F.); marion.kiechle@tum.de (M.K.); holger.bronger@tum.de (H.B.); julia.dorn@tum.de (J.D.); viktor.magdolen@tum.de (V.M.); 4 German Cancer Consortium (DKTK), Partner Site Munich, 81675 Munich, Germany; 5 German Cancer Research Center (DKFZ), 69120 Heidelberg, Germany

**Keywords:** miR-221, uPAR, triple-negative breast cancer, biomarker

## Abstract

Background: Triple-negative breast cancer (TNBC) is associated with poor prognosis and limited targeted treatment options. The urokinase plasminogen activator receptor (uPAR) contributes to tumor aggressiveness and may be regulated by microRNAs such as miR-221. This study aimed to evaluate the prognostic relevance of uPAR mRNA and miR-221 expression in TNBC. Methods: uPAR mRNA and miR-221 expression levels were quantified by real-time PCR in tumor tissues from 101 patients with TNBC. Associations with clinicopathological parameters and disease-free survival (DFS) were analyzed using univariate and multivariable Cox regression models. In silico analyses of publicly available datasets were performed for validation and, in addition, for further miR-221 target prediction. Results: In both univariate and multivariable analyses, high uPAR mRNA expression was associated with shorter DFS, whereas, in contrast, elevated miR-221 expression correlated with improved DFS. No inverse correlation between uPAR and miR-221 expression was observed, making a direct regulatory miR-221/uPAR axis in TNBC unlikely. Still, combined analysis revealed a pronounced additive prognostic effect, with high uPAR and low miR-221 expression identifying patients with the poorest DFS. These findings were supported by in silico analysis with publicly available patient data. Finally, other potential miR-221 targets were identified by applying in silico target prediction. Conclusions: uPAR and miR-221 represent independent prognostic markers in TNBC. Their combined expression provides additional prognostic value for disease-free survival and supports their potential relevance as biomarkers and therapeutic targets in TNBC.

## 1. Introduction

Breast cancer is one of the leading causes of cancer-related death among women worldwide. A woman born today has an approximately 1 in 8 lifetime risk of being diagnosed with breast cancer, accounting for about 16% of cancer-related deaths in women globally [1]. While early detection enables effective treatment in many cases, therapeutic options strongly depend on tumor subtype. Standard treatment strategies include surgery, radiotherapy, and systemic therapies. Molecular classification based on hormone receptor (HR) expression and human epidermal growth factor receptor 2 (HER2) amplification enables targeted therapies, such as endocrine therapy or HER2-directed agents.

Triple-negative breast cancer (TNBC), defined by the absence of estrogen receptor (ER) and progesterone receptor (PR), and low or absent HER2 expression, represents a particularly aggressive subtype. TNBC is characterized by high heterogeneity, early metastatic spread, and poor clinical outcome [2]. Due to the lack of established therapeutic targets, treatment options are limited mainly to chemotherapy, including platinum-, taxane-, and anthracycline-based regimens, as well as immune checkpoint inhibition with pembrolizumab. More recently, antibody–drug conjugates such as trastuzumab deruxtecan or sacituzumab govitecan and PARP inhibitors in BRCA1/2-mutated tumors have expanded therapeutic options [3,4]. Nevertheless, prognosis remains poor for many patients, highlighting the need for additional biomarkers to improve risk stratification and guide therapeutic decision-making [5].

The urokinase plasminogen activator receptor (uPAR) is a glycosylphosphatidylinositol-anchored cell surface receptor that plays a key role in extracellular matrix remodeling and cell migration. While uPAR is expressed at low levels in normal tissues, its expression is markedly increased in various malignancies, including breast cancer [6,7,8,9]. High uPAR expression has consistently been associated with aggressive tumor behavior and unfavorable prognosis [10]. Functionally, uPAR activity is regulated by a complex network involving proteolytic activation, interactions with extracellular matrix components, and modulation of intracellular signaling pathways [11]. In addition, uPAR expression itself can be regulated at multiple levels, including transcriptional and post-transcriptional mechanisms [12].

MicroRNAs (miRNAs) are short non-coding RNAs that regulate gene expression by promoting mRNA degradation or inhibiting translation [13]. In breast cancer, dysregulated miRNA expression has been linked to key oncogenic processes, including proliferation, epithelial–mesenchymal transition (EMT), immune escape, and therapy resistance [14,15,16]. Due to their cancer-specific expression patterns, miRNAs have emerged as promising prognostic and predictive biomarkers [17].

Among these, miR-221 has been described as an oncogenic miRNA in several malignancies [18]. In TNBC, miR-221 expression is significantly higher than in other breast cancer subtypes, both in cell lines and tumor tissues [19,20,21,22]. Functionally, miR-221 has been implicated in ER repression, thereby promoting the TNBC phenotype [23], and has been associated with enhanced proliferation, chemoresistance, EMT induction, and metastatic potential [21,24,25,26,27,28,29,30]. Paradoxically, several clinical studies have reported an association between high miR-221 expression and improved outcome in TNBC, suggesting a context-dependent biological function [31,32,33].

Experimental studies have proposed a regulatory interaction between miR-221 and uPAR, either through direct targeting of uPAR transcript variants or indirect regulation via intermediate signaling pathways [34]. However, whether this proposed miR-221/uPAR axis is clinically relevant in human TNBC remains to be studied.

The present study aimed to (i) evaluate the prognostic significance of uPAR and miR-221 expression in a well-characterized TNBC cohort, (ii) assess whether a clinically meaningful relationship between miR-221 and uPAR exists in vivo, and (iii) determine whether combined analysis of both markers improves prognostic stratification. To strengthen the clinical relevance of our findings, we additionally performed validation using publicly available datasets and in silico target analyses.

## 2. Materials and Methods

### 2.1. Patients

The study cohort comprised 101 patients diagnosed with TNBC between 1988 and 2012 at the Department of Obstetrics and Gynecology, Klinikum rechts der Isar, Technical University of Munich (TUM). Tumor tissue was collected during surgery, examined by pathologists, and immediately stored in liquid nitrogen. All tumors were tested for estrogen receptor (ER), progesterone receptor (PR), and human epidermal growth factor receptor 2 (HER2) expression as part of routine clinical diagnostics.

Classification as TNBC was defined by low ER and PR expression (immunohistochemical staining <1% for ER and <5% for PR) and low or absent HER2 expression (immunohistochemical score 0 or 1+). Equivocal cases with a score of 2+ were further analyzed for HER2 gene amplification by fluorescence in situ hybridization (FISH); cases without amplification were also classified as HER2-low. The TNBC status of all samples was re-evaluated by pathologists in 2013. Only patients with primary breast cancer and without neoadjuvant chemotherapy were included in the study. Patients with evidence of secondary malignancies were excluded.

Most breast cancer samples were classified as invasive ductal carcinomas of grade 2 or 3. A minority of cases represented less frequent subtypes, such as medullary or lobular carcinomas. Seventy-two percent of patients received adjuvant chemotherapy (anthracycline- or cyclophosphamide-based) following surgery, which included either mastectomy or breast-conserving surgery with axillary sentinel lymph node biopsy or dissection. At the time of surgery, no distant metastases were detected.

The median follow-up time was 72 months (range: 3–269 months) for disease-free survival (DFS) and 81 months (range: 4–286 months) for overall survival (OS). The median age at diagnosis was 60 years (range: 30–96 years). Tumor size ranged from 5 to 110 mm (median: 25 mm), and 45 patients had lymph node involvement. A summary of the patient characteristics can be found in Table S1.

The local Ethics Committee approved the analysis of biomarkers in tumor tissue from TNBC patients in accordance with the Declaration of Helsinki. Written informed consent was obtained from all participants.

### 2.2. Real-Time Polymerase Chain Reaction

Total RNA and microRNA were isolated from fresh-frozen tumor tissue using the AllPrep DNA/RNA/miRNA Universal Kit (Qiagen, Hilden, Germany) and the automated QIAcube system (Qiagen). Reverse transcription was performed using the miScript II RT Kit (Qiagen) following spectrophotometric assessment of RNA concentration and quality. A ratio of 260/280 and 260/230 below two was considered pure enough for processing. RNA integrity was evaluated by Agarose separation and visual detection of ribosomal RNA bands.

Quantification of miR-221 and SNORD68 (used as a housekeeping small non-coding RNA for normalization) was performed using the miScript Primer Assay (Qiagen) and the miScript SYBR Green PCR Kit (Qiagen, 218076). Specific primers for miR-221-3p (MS00003857) and SNORD68 (MS00033712) were applied.

Expression of uPAR and the housekeeping gene HPRT1 was analyzed using TaqMan Gene Expression Assays (Thermo Fisher Scientific, Waltham, MA, USA). The primers used were uPAR (Hs00958880_m1, detecting all four annotated uPAR isoforms: NP_002650, NP_001005376, NP_001005377, NP_001287966) and HPRT1 (Hs02800695_m1), together with Brilliant III Ultra-Fast Probe Low ROX qPCR Master Mix (Agilent, Santa Clara, CA, USA). The gene of interest and the housekeeping gene were measured on the same plate to minimize inter-assay variability. Total RNA from the TNBC cell line MDA-MB-231 was included in each run as a calibrator.

Reaction efficiency and sensitivity were determined using standard dilution series. Relative RNA expression levels were calculated using assay-specific threshold values and normalized to SNORD68 (for miR-221) or HPRT1 (for uPAR mRNA) using the 2^−ΔΔCT^ method. Stability of SNORD68 expression in our TNBC cohort has been determined by geometric mean evaluation using the RefFinder platform [35].

Each patient-derived sample was measured in triplicates following internal quality control. Criteria were defined, and samples were excluded if one or more of the following applied: standard deviation between duplicate measurements >47.1%; CT value of the housekeeping gene >35; or a 2^−ΔΔCT^ error exceeding 30%.

### 2.3. miR-221 Target Prediction and Gene Enrichment Analysis

Putative target genes of miR-221 were identified using three independent miRNA target prediction databases: miRDB, miRWalk, and TargetScan. Only genes predicted by all three databases were considered for further analysis in order to increase confidence and reduce false-positive results. The intersection of the three datasets was determined, and the resulting overlapping gene list was used for downstream analyses.

The top 66 overlapping target genes, ranked by prediction scores and database consistency, were subjected to functional enrichment analysis. Gene ontology (GO) enrichment was performed to assess significantly overrepresented biological processes, molecular functions, and cellular components. Pathway enrichment analyses were conducted using Kyoto Encyclopedia of Genes and Genomes (KEGG) and Reactome pathway databases. For all enrichment analyses, *p*-values were adjusted for multiple testing using the Benjamini–Hochberg false discovery rate (FDR) method, and adjusted *p*-values < 0.05 were considered statistically significant.

For downstream expression and correlation analyses, the top 50 target genes were selected based on enrichment relevance and biological significance. These genes were analyzed in the METABRIC breast cancer cohort. A detailed list of the used cases can be found in Table S2. Expression data were used to assess the relationship between miR-221 and its predicted target genes. Correlation analyses were performed using Spearman’s rank correlation coefficient, as expression values did not follow a normal distribution. Correlation strength and significance were evaluated for each gene, and results were used to identify potential regulatory associations between miR-221 and its predicted targets.

### 2.4. Statistics

Univariate and multivariable Cox regression analyses were performed to assess the association between RNA expression levels and clinical parameters with patient survival. Results are presented as hazard ratios (HRs) with 95% confidence intervals (95% CI). Kaplan–Meier survival curves were generated and compared using the log-rank test. An optimized cut-off was selected using Kaplan–Meier-based stratification to maximize separation between survival curves.

Associations between RNA expression levels and clinicopathological parameters were analyzed using the chi-square test. Correlations between continuous variables were evaluated using Spearman’s rank correlation coefficient. In silico analyses were performed using the KM Plotter and cBioPortal databases. Statistical analyses were conducted using SPSS software version 20.0 (SPSS Inc., Chicago, IL, USA). A *p*-value < 0.05 was considered statistically significant.

## 3. Results

### 3.1. Intratumoral Expression of miR-221 and uPAR mRNA in TNBC and Correlation with Clinical Parameters

miR-221 and uPAR mRNA expression levels showed broad interindividual variability across TNBC samples. miR-221 expression ranged from 0.019 to 13.026 (median 3.219), while uPAR mRNA levels ranged from 0.251 to 18.507 (median 0.273). miR-221 and uPAR mRNA were positively correlated (r_s_ = 0.325, *p* = 0.001), analysis of publicly available TNBC data revealed a similar positive correlation (r_s_ = 0.207, *p* = 0.001). The lack of an inverse correlation between miR-221 and uPAR mRNA levels speaks against a direct targeting of uPAR mRNA by miR-221 and subsequent degradation in TNBC (Figure S1).

For subsequent analyses, miR-221 expression was dichotomized at the median and uPAR expression at the upper tertile. No significant associations were observed between miR-221 or uPAR expression and clinicopathological parameters including age, tumor size, lymph node status, or administration of chemotherapy (Table 1; Figure S2), indicating that expression levels are independent of established clinical risk factors.

### 3.2. Association of miR-221 and uPAR mRNA Expression Levels with Survival in Univariate Regression Analysis

The prognostic relevance of miR-221 and uPAR mRNA expression, as well as clinicopathological parameters, was evaluated using univariate Cox regression analysis in the TNBC cohort. Among the clinical variables, older age (>60 vs. ≤60 years) and positive lymph node status were associated with reduced DFS and OS, whereas chemotherapy administration was associated with improved DFS and OS (Table 2).

High miR-221 expression was associated with prolonged DFS (HR: 0.50; 95% CI: 0.25–1.00; *p* = 0.046), but showed no significant association with OS (Table 2).

**Figure 1:**
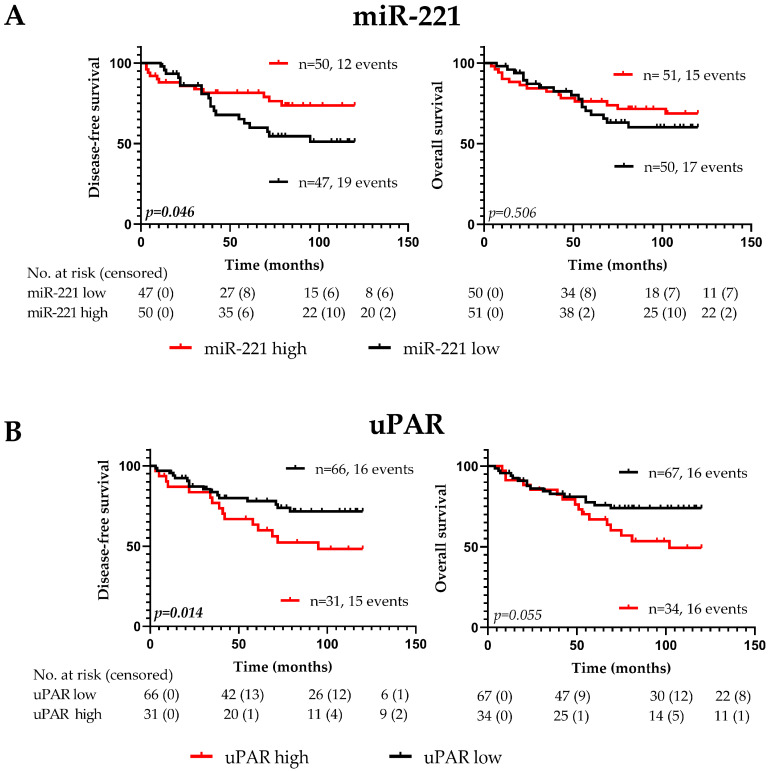
Impact of miR-221 (**A**) and uPAR mRNA (**B**) expression in primary tumor tissue on disease-free and overall survival depicted as Kaplan–Meier survival curves. The respective RNA expression was dichotomized into low (black) and high (red) expression by the median for miR-221 and the 66th percentile for uPAR, respectively.

**Figure 2:**
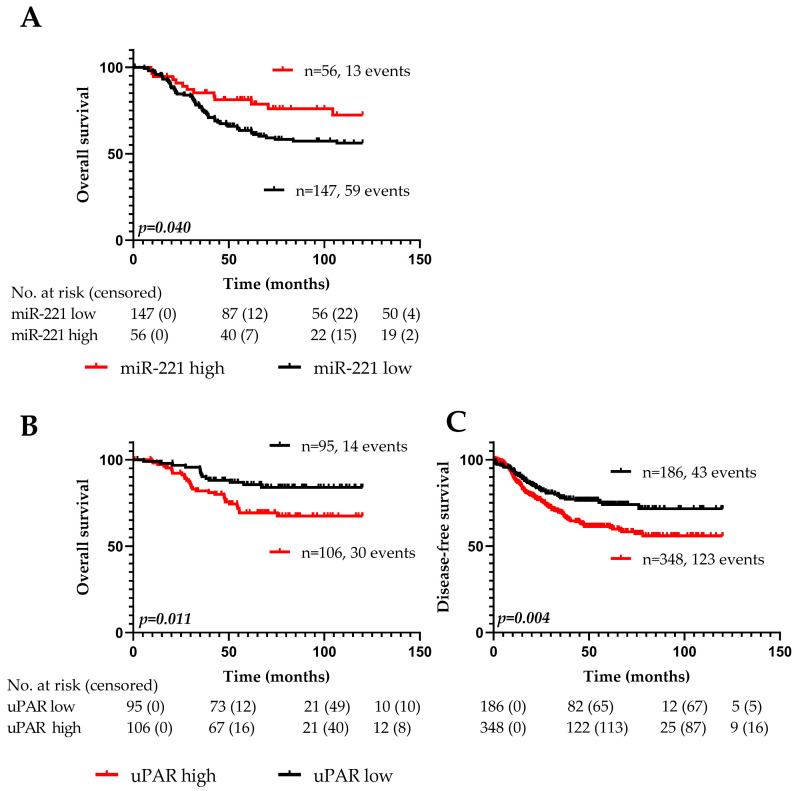
In silico analysis showing the relation of miR-221 and uPAR mRNA expression levels with patient prognosis. (**A**) Association of miR-221 expression levels with overall survival. Association of uPAR mRNA expression with DFS (**B**) and OS (**C**) of TNBC patients. Both datasets were derived from KM Plotter data [36].

The favorable impact of miR-221 on DFS was confirmed by Kaplan–Meier analysis (*p* = 0.046; Figure 1A). In silico analysis using publicly available datasets [36] further revealed an association between high miR-221 expression and prolonged OS (*p* = 0.040; Figure 2A). Information on DFS was not available in this dataset. Taken together, these findings suggest that elevated miR-221 expression represents a favorable prognostic marker in TNBC.

In contrast, elevated uPAR mRNA expression was significantly associated with shorter DFS (HR: 2.24; 95% CI: 1.15–4.35; *p* = 0.017) and showed a trend towards reduced OS (HR: 1.95; 95% CI: 0.97–3.89; *p* = 0.060) in univariate Cox regression analysis (Table 2). Kaplan–Meier survival analyses confirmed these findings, demonstrating significantly shorter DFS (*p* = 0.014) and a trend towards reduced OS (*p* = 0.055) in patients with high uPAR expression (Figure 1B). Importantly, analysis of an independent public dataset [36] also revealed a significant association between elevated uPAR mRNA levels and both shorter DFS (*p* = 0.004) and OS (*p* = 0.011) (Figure 2B,C). Collectively, these data indicate that high uPAR mRNA expression represents an unfavorable prognostic marker in TNBC.

### 3.3. Association of miR-221 and uPAR mRNA Expression Levels with Survival in Multivariable Cox Regression Analysis

In a multivariable Cox regression model adjusting for age, tumor size, lymph node status, and chemotherapy, miR-221 expression remained an independent predictor of prolonged disease-free survival (HR 0.316, 95% CI 0.146–0.683, *p* = 0.003). Conversely, high uPAR expression was significantly and independently associated with reduced DFS (HR 3.044, 95% CI 1.405–6.595, *p* = 0.005). The same tendencies for both markers were also found, with a borderline significant association with OS.

These findings indicate that miR-221 and uPAR provide prognostic information beyond established clinicopathological risk factors (Table 3).

### 3.4. Associations Between miR-221 and uPAR mRNA Levels and Prognostic Value of Their Combined Expression

Given the opposing prognostic effects of miR-221 and uPAR, we next assessed their combined prognostic value. Patients with high uPAR and low miR-221 expression exhibited the poorest clinical outcome, whereas patients with low uPAR and high miR-221 expression showed the most favorable prognosis. Intermediate outcomes were observed in patients with concordant expression patterns.

**Figure 3:**
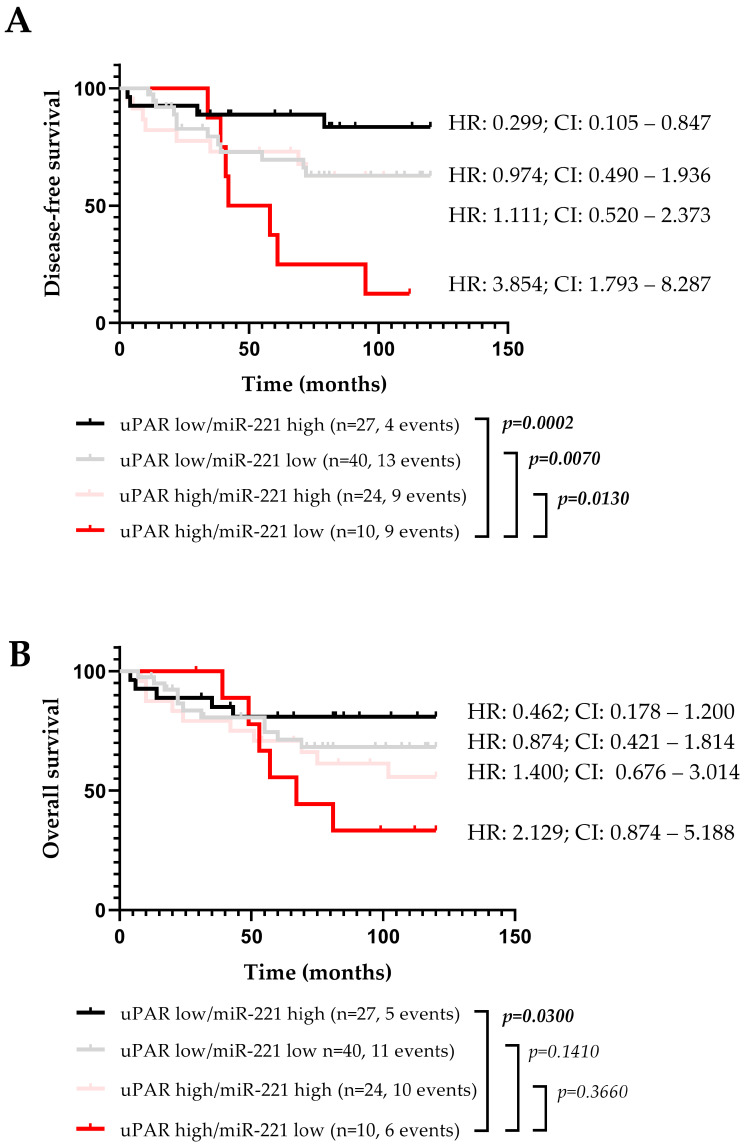
Subgroup analysis of combinations of expression levels of miR-221 and uPAR with respect to their impact on disease-free (**A**) and overall survival (**B**). *p*-values were calculated by a log-rank test of each layer separately.

This combined analysis demonstrated a striking additive prognostic effect of both markers and allowed improved risk stratification compared to either biomarker alone (Figure 3; Table S3).

### 3.5. miR-221 Target Prediction in Breast Cancer Tissue

**Figure 4:**
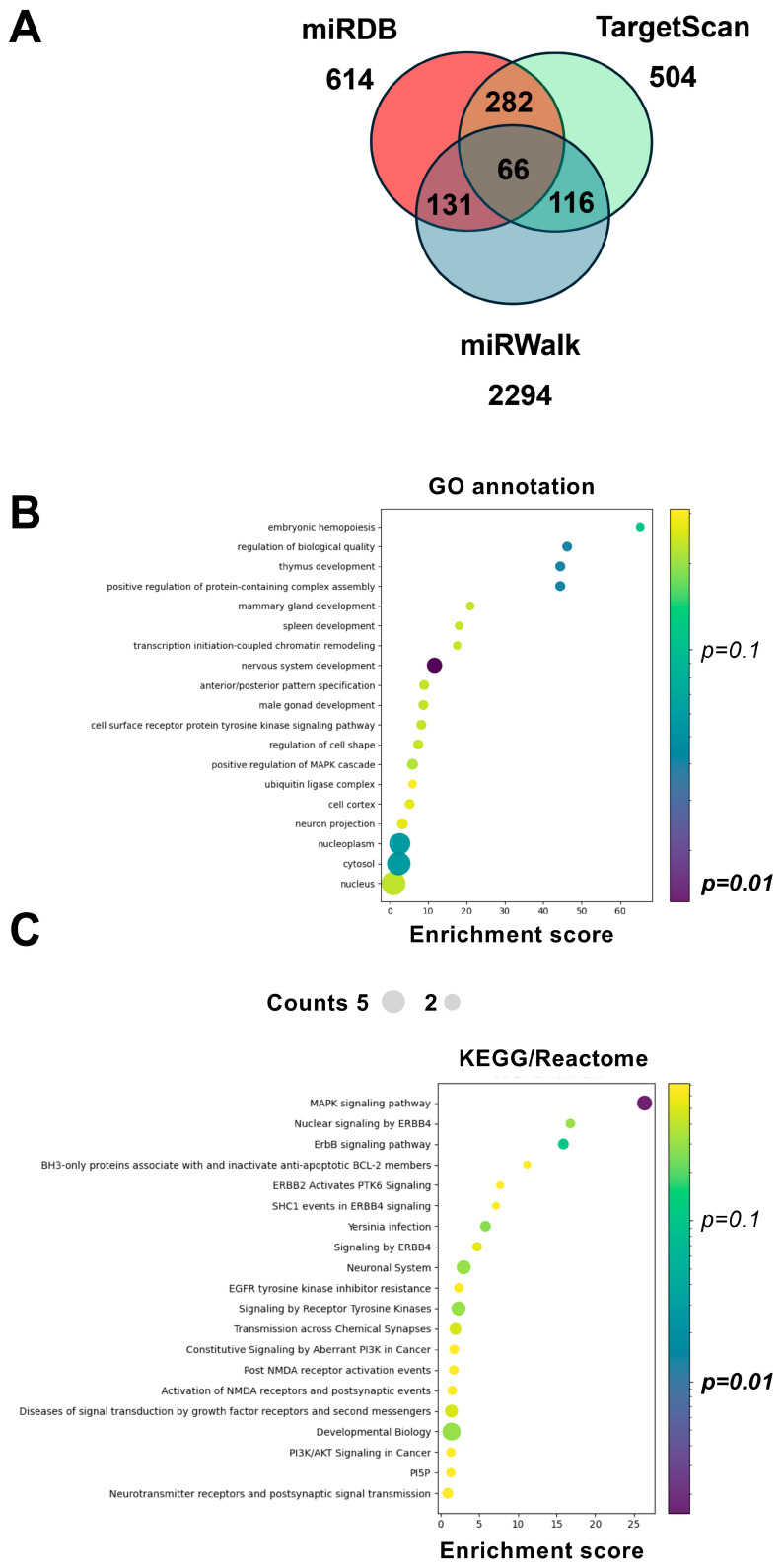
Target prediction of miR-221. (**A**) Three different target prediction tools (TargetScan, miRWalk, and miRDB) were used to identify potential target genes of miR-221. All obtained datasets were analyzed for overlap and concordance. The commonly identified target genes were subsequently subjected to pathway enrichment analysis using (**B**) Gene Ontology (GO) annotations (Cellular Component, Molecular Function, and Biological Process) as well as (**C**) KEGG and Reactome databases. The enrichment score combines fold enrichment and adjusted *p*-value. The number of annotated genes is represented by the size of the circles, while the color indicates the significance level of each term.

Since the initially hypothesized inverse regulation of uPAR by miR-221 was not evident in either our cohort or the publicly available TNBC datasets, we performed in silico target prediction using three independent databases (TargetScan, miRDB, and miRWalk) to identify other potential miR-221 targets in TNBC. In a first comprehensive approach, we screened for overlapping predicted targets across all three databases, yielding 66 potential miR-221 target genes (Figure 4A).

To further strengthen the relevance of these predicted interactions in TNBC, we analyzed data from the Metabric BRCA cohort. miR-221 expression levels and corresponding target gene mRNA expression data from TNBC samples were extracted, and potential correlations between miR-221 and its predicted targets were assessed. Moreover, numerous target mRNAs exhibited inverse correlations with miR-221 expression, suggesting possible negative regulation by miR-221 (Figure S3).

To link these candidate target genes to biological function, we performed gene ontology (GO) and pathway enrichment analyses. The top 20 enriched terms from both GO annotation and Kyoto Encyclopedia of Genes and Genomes (KEGG)/Reactome pathway analyses are presented in Figure 4 B and C. The most significantly enriched processes include tissue development and hematopoiesis, alongside marked involvement in pro-apoptotic signaling pathways, notably BCL-2 and MAPK. These pathways are well recognized for their roles in breast cancer pathobiology and cellular stress responses.

## 4. Discussion

In the present study, we demonstrate that miR-221 and uPAR expression in TNBC exhibit complementary prognostic potential. While high uPAR expression, consistent with its proposed role in tumor invasion and extracellular matrix remodeling, was associated with poor prognosis, elevated miR-221 expression was associated with significantly prolonged disease-free survival in patients with triple-negative breast cancer. This association remained significant in multivariable analysis, supporting miR-221 as an independent prognostic marker in this tumor entity. These findings suggest that both markers reflect distinct, partly overlapping processes in TNBC and may support combined risk stratification.

At first glance, this observation appears counterintuitive, as miR-221 has frequently been described as an oncogenic microRNA in experimental systems of different cancer entities including breast cancer. In vitro studies have linked miR-221 to increased proliferation [21,25], epithelial–mesenchymal transition [27,29], chemoresistance [26,30], and evasion of apoptosis [37]. However, the biological effects of miR-221 are highly context-dependent and strongly influenced by tumor type, molecular background, and microenvironmental factors [38]. This strong context dependency is also reflected in the available clinical literature. While some studies have identified miR-221 as a favorable prognostic marker for disease-free survival in TNBC, other studies and bioinformatic analyses report an unfavorable association, particularly for miR-221 in basal-like subtypes [39,40].

Several biological mechanisms may explain the favorable prognostic association observed in our TNBC cohort. The first aspect encompasses the direct targets of miR-221, including factors such as poly(ADP-ribose) polymerase 1 (PARP1), that increase sensitivity to genotoxic stress and, consequently, influence the response to pharmacological treatment [41,42]. In a TNBC-based study, the authors demonstrated a direct relationship between low miR-221 expression, increased PARP1 expression, and shortened disease-free survival [31]. Beyond this regulatory layer, PARP1 overexpression alone has been associated with a more aggressive tumor phenotype and unfavorable prognosis [43], supporting the relevance of a miR-221-high/PARP1-low profile as a marker of improved prognosis.

Moreover, miR-221 modulates immune and vascular signaling, including suppression of NF-κB-dependent pathways via A20 and inhibition of endothelial migration and tube formation [44,45]. Taken together, these findings suggest that miR-221 may exert tumor-suppressive effects in vivo despite its oncogenic behavior in certain experimental settings.

In line with the established role of uPAR in invasion, proteolysis, and signaling crosstalk [46]. uPAR remains a consistent marker of aggressiveness in our analysis. uPAR is not only relevant as a prognostic marker but also represents a promising translational target. uPAR-targeted imaging approaches have been shown to be safe and capable of tumor detection in first-in-human studies [47]. Furthermore, prospective data in early breast cancer indicate that, despite limited staging performance, uPAR positivity in primary tumors may support the development of further theranostic strategies. In addition, the preclinical development of uPAR-targeting antibodies, such as huATN-658, offers promising avenues for future therapies, particularly in TNBC [48].

Besides analyzing the prognostic relevance of both uPAR mRNA and miR-221 in TNBC, a central aim of this study was to assess whether a direct regulatory relationship between miR-221 and uPAR, resulting in lower uPAR mRNA levels in miR-221 high-expressing tumor tissues, can be identified in clinical TNBC samples. Our analyses—supported by in silico prediction tools—did not reveal an inverse correlation between miR-221 and uPAR expression in tumor tissue arguing against a strong or uniform direct regulatory interaction between both molecules at the mRNA level in the clinical setting. Still, the two biomarkers exhibited opposing prognostic associations, likely reflecting the complexity of miRNA–target interactions in vivo, which exhibit dynamic, stoichiometric, and cell state-dependent regulation, resulting in effects that are not always captured by bulk RNA data [49]. Therefore, the absence of a direct inverse correlation does not exclude indirect effects of miR-221 on uPAR-related pathways. miR-221 may instead influence tumor progression through alternative targets affecting processes such as extracellular matrix remodeling, cell adhesion, or proteolysis, which could explain why combined assessment of miR-221 and uPAR provides additive prognostic information.

Together, these findings suggest that miR-221 and uPAR represent biologically and clinically distinct, yet complementary, biomarkers in TNBC. Rather than reflecting a simple linear regulatory relationship, their inverse prognostic associations likely arise from their involvement in different tumor-relevant pathways, underscoring the complexity of post-transcriptional regulation in aggressive breast cancer subtypes.

To further investigate the biological role of miR-221, we conducted in silico target prediction followed by pathway enrichment analysis. Integration of multiple prediction algorithms identified 66 consensus target genes. All except three (ANKIB1, ATAD2B, and FMR1) were expressed in TNBC tissues according to METABRIC data. Numerous of these targets showed inverse correlations with miR-221 expression, consistent with a post-transcriptional regulation of these mRNAs by miR-221.

Functional annotation of the predicted targets revealed significant enrichment of pathways involved in key oncogenic processes, including MAPK, PI3K/AKT, EGFR, and apoptosis-related signaling. These pathways are known to play central roles in breast cancer progression, therapy resistance, and cellular stress responses [50,51,52].

A potential explanation for the favorable prognostic association of miR-221 may lie in its interaction with receptor tyrosine kinase signaling pathways. Several experimental and computational studies suggest that miR-221 regulates components of the EGFR signaling network. EGFR represents a central upstream activator of both the MAPK and PI3K/AKT pathways, which drive proliferation, migration, and survival in many cancers, including TNBC [53]. If miR-221 reduces EGFR expression or signaling activity, this could attenuate downstream oncogenic signaling and thereby limit tumor progression.

The MAPK cascade regulates proliferation, differentiation, migration, and various stress responses [54]. In TNBC, MAPK activity is often increased and contributes to epithelial–mesenchymal transition (EMT), metastatic potential, and therapy resistance [55,56]. Direct targeting of several receptor tyrosine kinases linked to MAPK pathway topology has been reported. In particular, PAK1 harbors a direct 3′-UTR binding site for miR-221 and can modulate MAPK activity [57]. The relationship appears to be context-dependent: in some TNBC settings, miR-221 may dampen MAPK-driven aggressiveness, whereas in others it may reinforce MAPK signaling. MAPK effectors such as c-Jun and 14-3-3ζ can upregulate miR-221, creating a positive feedback loop, and miR-221-mediated downregulation of CDKN1B/p27 may further promote proliferation [37,58].

Following this, miR-221 has also been suggested to regulate components of the PI3K/AKT pathway, a central signaling axis controlling cell survival and resistance to apoptosis. For example, miR-221 can target PTEN, a negative regulator of PI3K signaling, potentially leading to increased AKT activation in certain experimental systems [59]. However, the functional consequences of this interaction appear to be highly context-dependent. In TNBC, where PI3K signaling is frequently altered through additional genetic or epigenetic mechanisms [60], the impact of miR-221 on this pathway may be less dominant or may be counterbalanced by effects on other signaling networks.

miR-221 also inhibits intrinsic apoptotic signaling by targeting multiple pro-apoptotic BCL2-family members, including BIM (BCL2L11) and BMF in hepatocellular, ovarian and breast cancer [61,62,63]. Consistent with this, both genes emerged in our miR-221 target prediction approach and were inversely correlated with miR-221 in the TNBC Metabric dataset. Repression of these genes may reduce apoptotic responsiveness and promote resistance to cellular stress and cytotoxic stimuli [64]. These findings support miR-221 as a pleiotropic regulator of interconnected signaling pathways rather than a single dominant target. By modulating EGFR, MAPK, and PI3K signaling, miR-221 may reshape oncogenic networks in a context-dependent manner, providing one possible explanation for its association with improved clinical outcome in our cohort.

Although the prognostic relevance of both markers has been demonstrated in this study, several limitations should be considered when interpreting the biological interaction between miR-221 and uPAR. First, bulk tumor mRNA analysis lacks spatial resolution between cellular compartments. Second, patients in this study were enrolled between 1988 and 2012, and did not receive neoadjuvant chemotherapy. In addition to the long follow-up period, treatment regimens have evolved since then and no longer reflect the current standard of care. However, the limited use of systemic treatment may also provide clearer insight into the intrinsic biological roles of uPAR and miR-221 expression, particularly with respect to their prognostic potential. Third, the main conclusions of this study are based on correlation analyses, which provide robust estimates of clinical relevance but do not permit detailed mechanistic conclusions. We performed several in silico analyses to generate hypotheses regarding potential modes of interaction; however, functional studies will be required to validate the mechanistic roles of miR-221 and uPAR in TNBC.

The results of this study align with the need for multivariable prognostic models, especially in TNBC. One potential approach is the establishment of a multimarker panel, as previously suggested, incorporating miR-221, miR-1305, miR-4708, and RMDN2, which has the capacity to stratify TNBC risk groups [33]. Given the stability of miRNAs in liquid biopsies, particularly in blood-derived extracellular vesicles [65], implementation in clinical routine may be feasible once appropriate validation and decision models have been established. Beyond prognostic value, miR-221 may also support therapeutic decision-making.

First, with regard to overall chemosensitivity and response to PARP inhibitors, the direct interaction between miR-221 and PARP1 suggests that assessing miR-221 expression in TNBC patients could provide additional predictive value for PARP inhibitor-based therapies. Second, the expanding landscape of PI3K/AKT/mTOR-targeted therapies may also be linked to miR-221 expression, given its multiple interaction nodes within these pathways. Targeted therapeutic strategies depend on appropriate biomarker selection [66], and miR-221 may help to refine patient stratification for these next-generation therapies.

Our findings support miR-221 as a clinically relevant signal in TNBC and suggest that strand-specific miR-221-3p, in combination with its target genes and pathway context (e.g., PARP1/DNA repair, PTEN/PI3K, and MAPK signaling), may contribute to both prognostic and potentially predictive patient stratification.

In parallel, the development of standardized serum/plasma and exosomal miRNA assays represents a promising noninvasive translational pathway. Overall, combining functional validation with liquid-biopsy profiling may help resolve the context-dependent role of miR-221 and facilitate the transition from associative findings to clinically actionable biology.

## 5. Conclusions

This study demonstrates that uPAR and miR-221 represent independent and biologically distinct prognostic biomarkers in triple-negative breast cancer. Our data do not support the existence of a dominant direct miR-221/uPAR regulatory axis in clinical TNBC specimens.

While elevated uPAR expression identifies patients with aggressive disease and poor prognosis, high miR-221 expression is associated with a favorable patient outcome. Combined assessment of both markers provides additive prognostic information and may improve risk stratification in TNBC.

These findings highlight the importance of validating mechanistic hypotheses in clinically relevant patient cohorts and underscore the complexity of miRNA-mediated regulation in human cancer. From a clinical perspective, the combined assessment of uPAR and miR-221 may allow improved risk stratification beyond conventional clinicopathological parameters. Given the availability of uPAR-targeting approaches and the emerging role of miRNA-based therapeutics, these findings may have translational implications for patient stratification and future therapeutic development.

Future studies should focus on validating these observations in independent cohorts and exploring the therapeutic implications of uPAR- and miR-221-associated signaling pathways in the context of modern targeted and immune-based therapies.

## Figures and Tables

**Table 1:** Association of relative miR-221 and uPAR mRNA expression levels and clinicopathological parameters in patients afflicted with triple-negative breast cancer.

Clinicopathological Parameters	uPAR mRNA Low/High ^a^	miR-221 Low/High ^a^
**Age**	*p* = 0.291	*p* = 0.123
≤60 years	37/15	42/10
>60 years	30/19	33/16
**Lymph node status**	*p* = 0.178	*p* = 0.417
N0	40/15	43/12
N+	27/18	32/13
**Tumor size**	*p* = 0.233	*p* = 0.452
≤20 mm	19/6	10/15
>20 mm	44/26	53/17
**Chemotherapy**	*p* = 0.360	*p* = 0.499
no	20/7	12/15
yes	47/26	38/35

^a^ Chi-square test. Due to missing values, samples do not always add up to 101; Cut-off point: miR-221 = median; uPAR mRNA = 66th percentile.

**Table 2:** Clinical outcome in triple-negative breast cancer patients: univariate Cox regression analysis including clinicopathological parameters and miR-221/uPAR mRNA expression.

Clinicopathological Parameters		DFS (120 Months)		OS (120 Months)	
	No. ^a^	HR (95% CI) ^b^	*p*	HR (95% CI) ^b^	*p*
**Age**			**0.016**		**0.001**
≤60 years	52	1		1	
>60 years	49	2.33 (1.17–4.64)		3.42 (1.58–7.42)	
**Lymph node status**			**0.046**		**0.015**
N0	55	1		1	
N+	45	2.00 (1.01–3.98)		2.49 (1.19–5.20)	
**Tumor size**			0.495		0.640
≤20 mm	25	1		1	
>20 mm	70	1.34 (0.58–3.08)		1.24 (0.50–3.06)	
**Chemotherapy**			**0.040**		**0.020**
no	23	1		1	
yes	71	0.346 (0.17–0.72)		0.29 (0.13–0.61)	
**uPAR mRNA ^c^**			**0.017**		*0.060*
low	67	1		1	
high	34	2.24 (1.15–4.35)		1.95 (0.97–3.89)	
**miR-221 ^c^**			**0.046**		0.508
low	50	1		1	
high	51	0.50 (0.25–1.00)		0.79 (0.40–1.59)	

^a^ Missing follow-up values result in sample size values below 101. ^b^ HR: hazard ratio; CI: confidence interval. ^c^ Cut-off point: miR-221 = median; uPAR mRNA = 66th percentile. Significant values are indicated in bold, and trends towards significance are indicated in italics.

**Table 3:** Clinical outcome in triple-negative breast cancer patients: multivariable Cox regression analysis including clinicopathological parameters and miR-221/uPAR mRNA expression.

Clinicopathological Parameters		DFS (120 Months)		OS (120 Months)	
	No. ^a^	HR (95% CI) ^b^	*p*	HR (95% CI) ^b^	*p*
**Age**			0.574		*0.061*
≤60 years	48	1		1	
>60 years	46	1.267 (0.556–2.889)		2.562 (0.957–6.856)	
**Lymph node status**			0.100		**0.048**
N0	51	1		1	
N+	43	1.949 (0.880–4.319)		2.495 (1.009–6.172)	
**Tumor size**			0.684		0.543
≤20 mm	24	1		1	
>20 mm	70	0.812 (0.299–2.207)		0.702 (0.224–2.198)	
**Chemotherapy**			**0.002**		**0.017**
no	23	1		1	
yes	71	0.250 (0.104–0.599)		0.344 (0.143–0.826)	
**uPAR mRNA ^c^**			**0.005**		*0.059*
low	63	1		1	
high	31	3.044 (1.405–6.595)		2.315 (0.969–5.530)	
**miR-221 ^c^**			**0.003**		0.097
low	46	1		1	
high	48	0.316 (0.146–0.683)		0.492 (0.213–1.138)	

^a^ Missing follow-up values can result in sample values below 101. ^b^ HR: hazard ratio, CI: confidence interval. ^c^ Cut-off point: miR-221 = median, uPAR mRNA = 66th percentile. Significant values are indicated in bold, and trends towards significance in italics.

## Data Availability

Data supporting the findings of this manuscript are not publicly available due to personal data security and privacy reasons, but are available on request from the corresponding author, TD. The publicly available data sets were accessed via the KM Plotter (https://kmplot.com/analysis/index.php?p=service&cancer=breast (accessed on 12 February 2026, TNBC cases defined as negative for the ER, PR and HER2)) or the cBioPortal platform (https://www.cbioportal.org/study?id=brca_metabric, accessed 12 February 2026, TNBC cases were defined as negative for the ER, PR, and HER2). The used cases for in silico analysis including the raw data can be found in Table S2. The databases were accessed in January 2026.

## Supplementary Materials

The following supporting information can be downloaded at: https://www.mdpi.com/article/10.3390/pathophysiology33020029/s1, Figure S1. Correlation of miR-221 and uPAR mRNA expression in TNBC tumor tissue and publicly available data; Figure S2. Association of relative miR-221 (A) and uPAR mRNA (B) expression levels and clinicopathological parameters (tumor size, age, lymph node status, and chemotherapy treatment) in patients afflicted with triple-negative breast cancer. Significances have been determined by Mann–Whitney U analysis; Figure S3. Correlation analysis of miR-221 expression and predicted target mRNA expression; Table S1. Clinicopathological characteristics of the TNBC patients; Table S2. METABRIC IDs used for in silico analysis and mRNA-expression raw data; Table S3. Numbers at risk and censored events for subgroup survival analysis of combined miR-221 and uPAR mRNA expression.

## Abbreviations

The following abbreviations are used in this manuscript:

ADC	antibody–drug conjugate
BRCA	breast cancer susceptibility gene
CI	confidence interval
DFS	disease-free survival
DKFZ	German Cancer Research Center
DOI	Digital Object Identifier
EMT	epithelial–mesenchymal transition
ER	estrogen receptor
FISH	fluorescence in situ hybridization
GO	Gene Ontology
HER2	human epidermal growth factor receptor 2
HR	hazard ratio
HPRT1	hypoxanthine phosphoribosyltransferase 1
KEGG	Kyoto Encyclopedia of Genes and Genomes
KM	Kaplan–Meier
miRNA	microRNA
miR-221	microRNA-221
mRNA	messenger RNA
NF-κB	nuclear factor kappa B
OS	overall survival
PARP	poly(ADP-ribose) polymerase
PCR	polymerase chain reaction
PR	progesterone receptor
qPCR	quantitative polymerase chain reaction
RNA	ribonucleic acid
SNORD68	small nucleolar RNA 68
TCGA	The Cancer Genome Atlas
TNBC	triple-negative breast cancer
uPAR	urokinase plasminogen activator receptor
