# Supplementary figures and images for: High uPAR and Low miR-221 Expression Predict Poor Disease-Free Survival in Triple-Negative Breast Cancer
Source: Pathophysiology. 2026 Apr 22;33(2):29. doi: 10.3390/pathophysiology33020029 (PMC13108178; doi:10.3390/pathophysiology33020029)

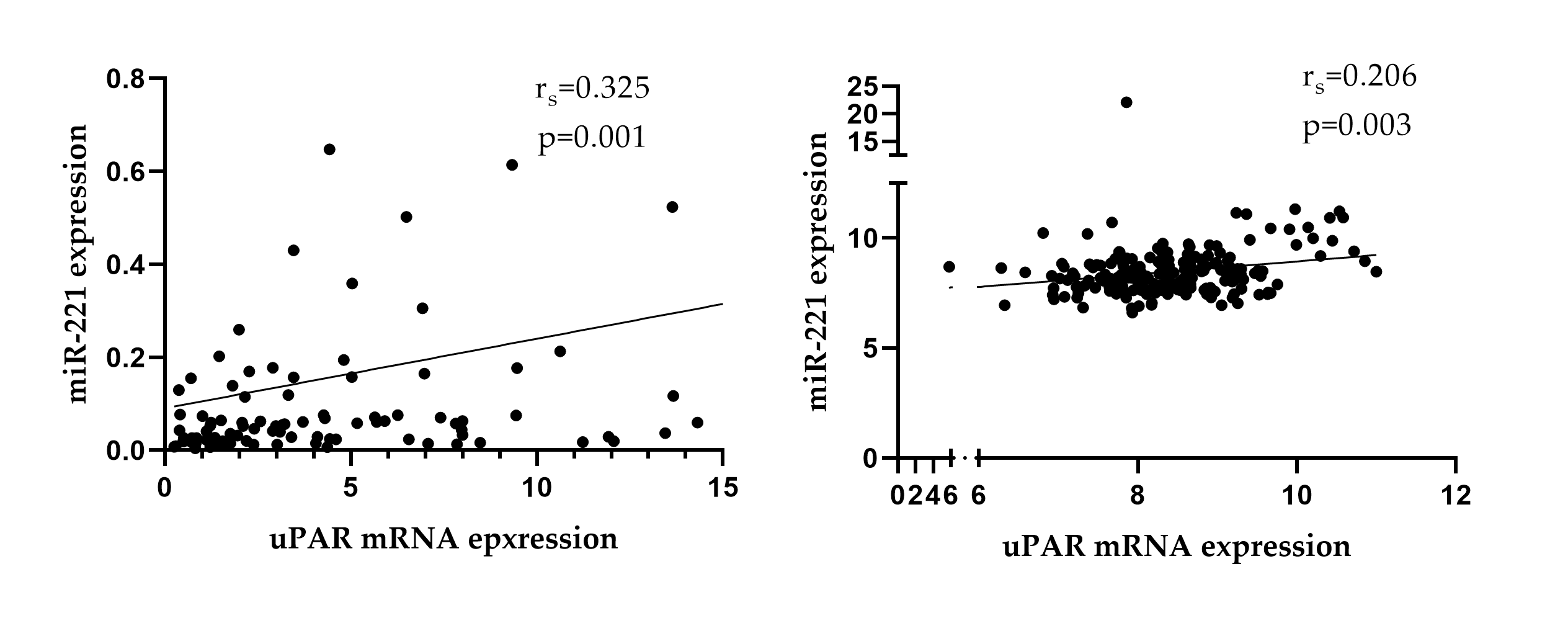

Supplement: Supplementary file 1 [file pathophysiology-33-00029-s001.zip › Figure S1.tif]

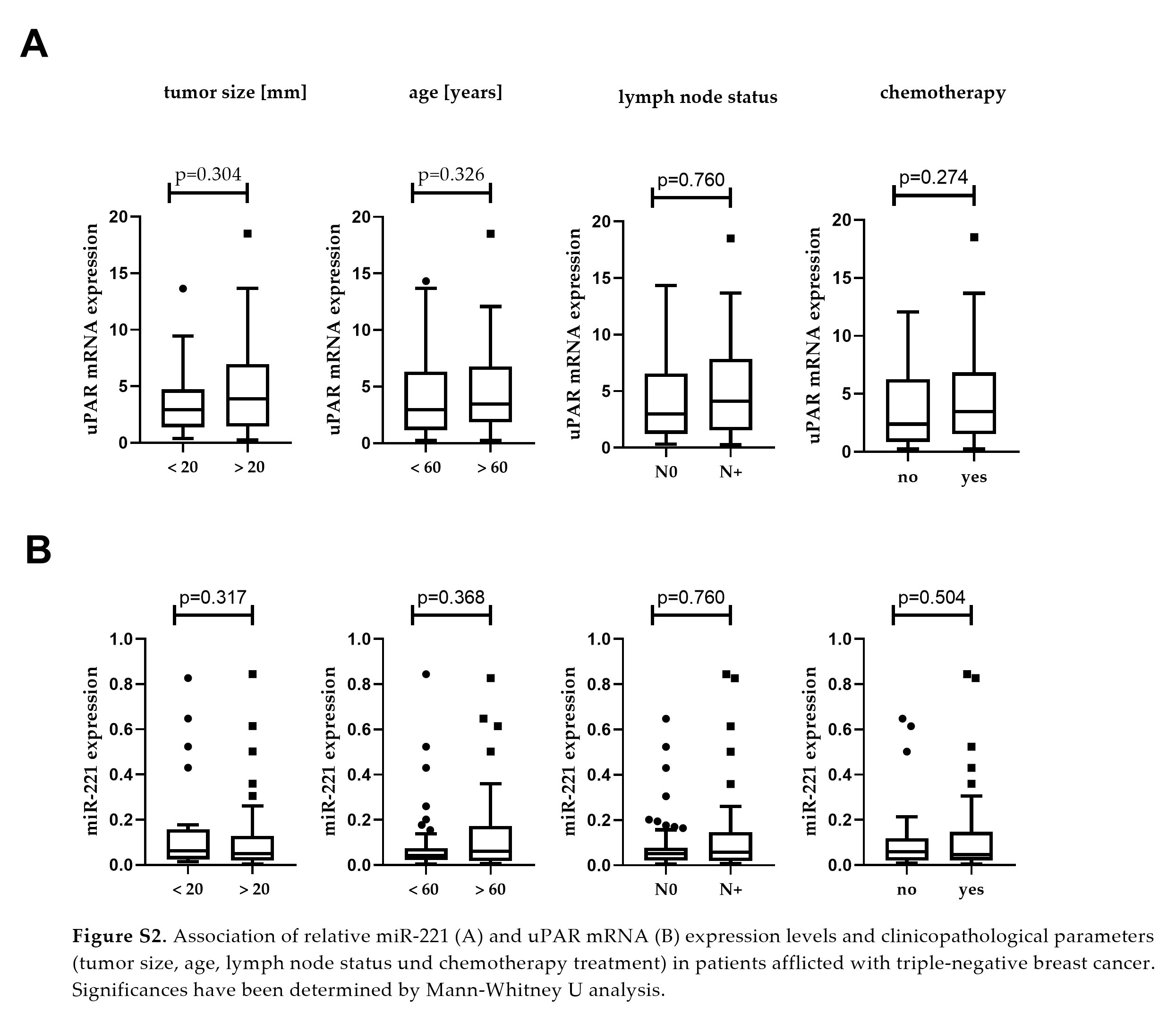

Supplement: Supplementary file 1 [file pathophysiology-33-00029-s001.zip › Figure S2.tif]

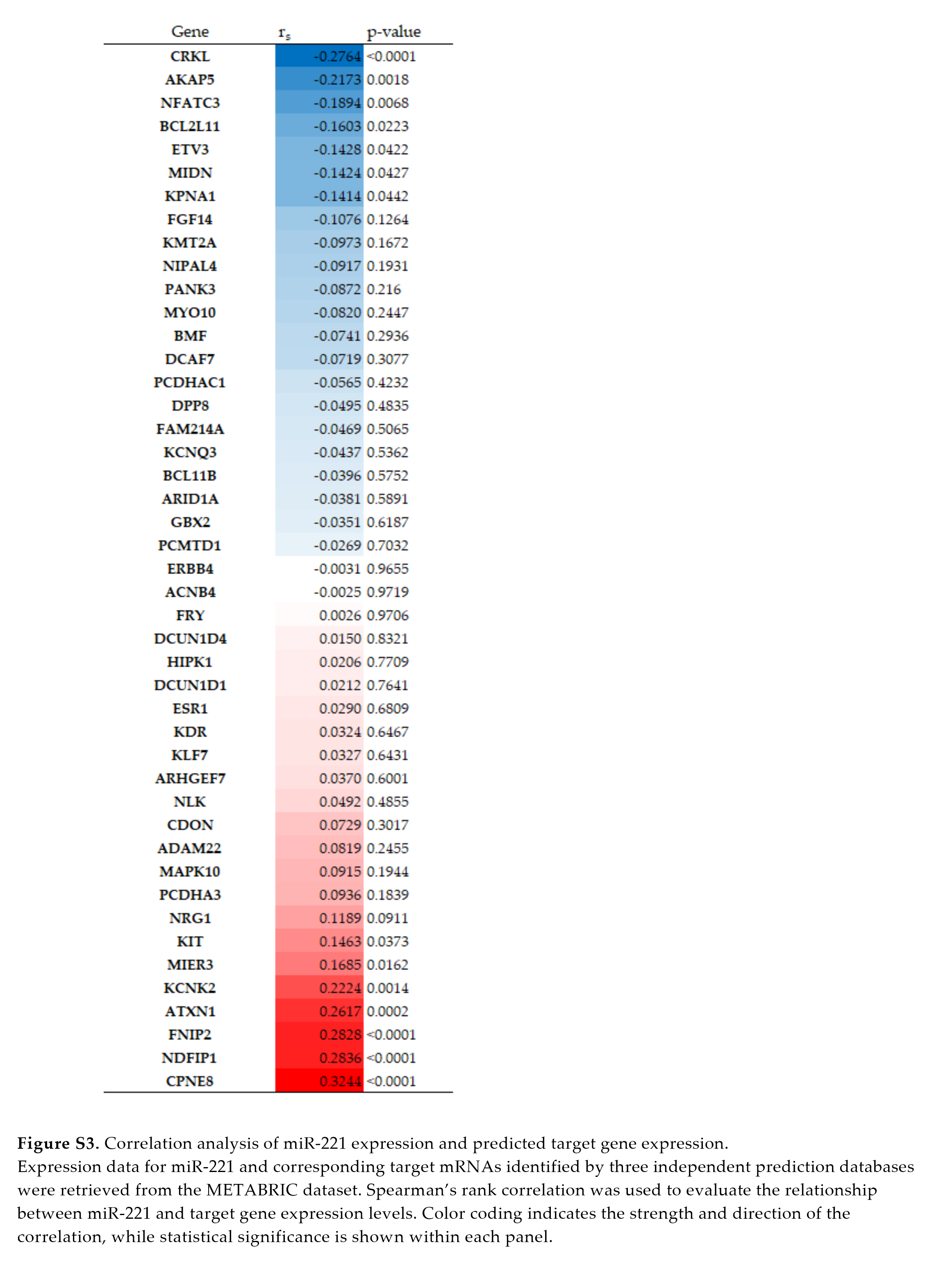

Supplement: Supplementary file 1 [file pathophysiology-33-00029-s001.zip › Figure S3.tif]
